# Application solutions of highway freight information systems based on quantum communication

**DOI:** 10.1038/s41598-024-52987-4

**Published:** 2024-02-01

**Authors:** Wenliang Tang, Yifan Gao

**Affiliations:** 1 East China Jiao Tong University, Nanchang City, Jiangxi Province China; 2 East China Jiao Tong University, Network Information Center, Nanchang City, China

**Keywords:** Computational science, Computer science

## Abstract

To improve the security of data transmission in the highway freight information system, this study is an application plan for the highway freight information system based on quantum communication. This solution is based on quantum communication technology to encrypt and transmit key sensitive data^[1]^; it realizes unified management of quantum keys through the quantum key cloud terminal and provides key services for the highway freight information system; it realizes access to the system through the quantum key cloud service platform. The secure use of mobile terminal quantum keys improves the overall security of the road freight information system. This scheme uses the quantum encryption key generated only once, effectively protecting the entire system's security. The quantum key management server and quantum key cloud platform defined in this plan manage terminals and quantum keys respectively, and jointly produce and distribute quantum keys with the help of other hardware facilities and software to provide secure transmission of key information.

## Introduction

The security of key data and sensitive data during the transmission process is very important. To protect the important data of the information system from being illegally obtained and then violently cracked, the traditional method can be to establish a private network or perform higher-level security on sensitive data. Encryption is used to ensure the security of these data as much as possible. Among them, the latter has a stronger ability to protect data security. However, with the continuous improvement of mechanical computing power, classical asymmetric encryption methods that rely on traditional computational complexity have great data security risks^1^. Due to the supercomputing parallel capabilities of quantum computers, no matter how complex it is to use traditional asymmetric encryption methods, all encryption methods can be violently cracked in a short period. Therefore, modern information systems urgently need to be upgraded to information systems that can withstand the violent calculations of quantum computers before the advent of complete quantum computers, that is, *quantum information systems*.

As a new type of secure communication technology, quantum communication is based on the physical properties of quantum non-replication and collapse once measured. It can be used to ensure the theoretical "*unconditional security*" of key transmission, that is, quantum keys cannot be cracked^1,2^. Under theoretical conditions, if a quantum is "*stolen*" or "*modified*", both the sender and the receiver can discover it and abandon the key information transmission after discovery, thereby ensuring the data security of the entire information system.

Currently, many scientists have notable research works on the successful implementation and integration of Quantum Key Distribution (QKD) in real network environments. In the second half of 2020, a research group led by Jian-Wei Pan from the University of Science and Technology in China demonstrated long-distance and secure measurement-device-independent quantum key distribution (MDI-QKD) over a free-space channel for the first time^3^. This work could pave the way to satellite-based MDI-QKD implementations. In 2023, another team from China conducted in-depth research on the quantum key distribution scheme with key recycling in the integrated optical network, providing profound guidance for the research in this article, which proposes a QKD-integrated four-layer optical network architecture^4^. The framework integrates QKD into the optical network, allowing multiple users to obtain the required key rate from the same QKD network infrastructure without deploying a dedicated QKD network^4^. The paper also introduces a quantum key recycling (QKR) mechanism, which improves the use efficiency of keys in optical networks^4^. During the same year, researchers led by the University of Geneva's Hugo Zbinden described a new QKD system, in which all components are integrated into chips except the laser^5^. This system achieves higher transmission speeds^5^. These studies represent significant advancements in the field of QKD, demonstrating its potential for real-world applications in secure communications.

At present, quantum communication equipment and systems that combine *quantum key distribution (QKD) technology*^6,7^, trusted relay technology, and optical fiber multiplexing technology have been pioneered in the military^8^, government^9^, education, and other industries. The road transportation industry is a major lifeline of our country's national economy^10^. Its various information systems integrate many important data. Once damaged, it will lead to data leakage and other security risks, which will cause huge losses to national security, economic development, and people's living and working in peace and contentment. With the development of road freight informatization, the network security situation it faces has become increasingly severe. Research on quantum communication technology is of great significance to ensure the safe operation of *road freight*. This article studies the application scheme of *highway freight information systems* based on *quantum communication* to improve the overall security of the information system in the highway freight industry in the future.

I will compare the *running time* and *success rate* of the quantum key distribution algorithm and ordinary RSA algorithm to express the feasibility of this project and to prove its theoretical feasibility. As for various problems that may be encountered in the real environment, this article will not consider them temporarily. These can be comprehensively evaluated before the project is implemented, including transmission distance. The potential reduction in quantum key distribution transmission efficiency is caused by quantum self-deflection and other factors. In the simulation, the success rate of Eve is set to 75%, and even in this case, the success rate of the QKD algorithm is close to 100% and almost completed instantly. The RSA algorithm can also perform effective prime factor decomposition, but it takes a long time. Decomposing 100 numbers in simulation takes 317 s, which is incomparable to the instantaneous completion of QKD. It seems that by the time RSA was successfully cracked, QKD had already completed the encryption and decryption of the signal. Quantum cryptography is a dimensionality reduction blow to classical cryptography, so a quantum-based logistics transportation cloud big data platform is feasible.

## Overview of quantum communications and related technologies

### Quantum Key Distribution (QKD) Technology

When people reduce the dimension of the observation world to the microscopic level, they will find that physical quantities will jump in steps with the smallest unit of variation, rather than continuously. This smallest unit of physical variation is the *Quantum*, and *Quantum* is not a specific kind of particle that refers to an indivisible basic individual, which is the tiny energy that makes up things, such as photons^11,12^. The *B92-QKD protocol* used in this article is a two-state quantum key distribution theory protocol based on photons. Quantum mechanics is the basic theory for studying the mechanical laws of the microscopic world, and its correctness has been gradually confirmed with the development of science. Research shows that the quantum state has "*intrinsic randomness*", that is to say, its randomness is the inherent characteristic of microscopic particles. Using this randomness, real random numbers can be generated, that is, "*true random numbers*" (or called "*intrinsic random numbers*"). *Quantum secure communication*^13^ is the highest-level secure communication technology that utilizes the indivisible and non-replicable characteristics of single-photon quantum states and is born in scenarios such as resistance to quantum computing cracking. It has unconditional security features that cannot be eavesdropped or deciphered^14^.

Quantum states are statistical concepts described by density operators, $\widehat{\rho }$

1 $$\widehat{\rho }=\sum_{i}|{\psi }_{i}\rangle \langle {\psi }_{i}|$$

The use of unitary transformation can ensure the conservation of eigenvalues in the quantum state transformation process, the expected satisfaction of any mechanical quantity $\widehat{Q}$ observation value is:

2 $$\langle \widehat{Q}\rangle =\sum_{i}{p}_{i}\langle {\psi }_{i}|\widehat{Q}|{\psi }_{i}\rangle ={T}_{r}\left({Q}_{i}\sum_{i}{p}_{i}|{\psi }_{i}\rangle \langle {\psi }_{i}|\right)={T}_{r}(\rho {Q}_{i})$$

where $|\psi \rangle$ is the wave function of the quantum state, represented as:

3 $$|\psi \rangle =\sum_{i=1}^{\infty }{Q}_{i}|{\psi }_{i}\rangle $$

If the projection operator $\widehat{Q}=|{\psi }_{i}\rangle \langle {\psi }_{i}|$ acts on $|\psi \rangle$, which means:

4 $$\widehat{Q}|\psi \rangle ={Q}_{i}|{\psi }_{i}\rangle $$

Then, randomly collapse quantum state $|\psi \rangle$ to the eigenstate $|{\psi }_{i}\rangle$, and obtain the observed eigenvalues for this time ${Q}_{i}$. But the observation caused disturbance, making quantum state ${|\Sigma \rangle }_{B}$ unclonable, that is:

5 $$\widehat{U}{|\psi \rangle }_{A}{|\Sigma \rangle }_{B}\ne {|\psi \rangle }_{A}{|\psi \rangle }_{B}$$

Quantum state $|\psi \rangle$ undergoes wave evolution according to the Schrödinger Equation, but its eigenvalue ${Q}_{i}$ reflects the mechanical properties of the observed instantaneous collapse state.

### B92-QKD protocol

The B92-QKD scheme^15^ is a modified scheme of BB84-QKD proposed by Bennett. Its security is guaranteed by the non-cloning theorem (security source). It transmits the *measurement results* instead of the measurement base (BB84 transmits the measurement base). The B92-QKD scheme is a *two-state protocol*^16^. Unlike the BB84-QKD protocol, which uses four non-orthogonal quantum states, B92-QKD only uses two non-orthogonal quantum states (Z state and X state) to complete random quantum key distribution. Since the qubits are used to satisfy the quantum non-cloning theorem, attackers cannot obtain valid quantum key information from the protocol^17^.

B92-QKD is a quantum key distribution protocol that allows two parties to securely exchange encryption keys over a public channel. The benefit of B92-QKD to the proposed network architecture is that it reduces the complexity and cost of the quantum devices, as well as the bandwidth requirements of the communication channel. B92-QKD only requires two non-orthogonal states to encode the key bits, which means that the sender and receiver only need single-photon sources and detectors, and no interferometers or phase modulators. Moreover, B92-QKD can tolerate higher bit error rates than other protocols, such as BB84, which makes it more robust against noise and eavesdropping attacks. Therefore, B92-QKD is a suitable protocol for implementing quantum cryptography in large-scale networks with limited resources and high-security demands.

**Figure 1:**
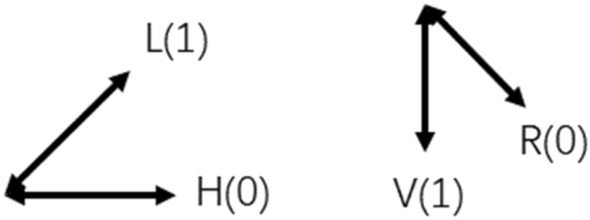
Schematic diagram of B92 photon quantum state encoding.

As shown in Fig. 1, the B92-QKD scheme encodes the photon quantum state of the horizontal ( ↔) direction |H > photon and the 45° (↗) direction |L > polarization into binary "0"; The quantum state of the photon which the vertical (↕) direction |V > photon and the |R > polarized photon in the 135° (↘) direction is encoded as “1”:

The sender Alice randomly selects |L > photons or |H > photons to modulate the quantum signal, so that the photons transmitted in the quantum channel are randomly in the fields of |L > photons and |H > photons, where |L > photons and |H > photons are expressed in the language of quantum mechanics. As follows:

6 $$|H> =|0>$$

7 $$|L> = \frac{|0>+|1>}{\sqrt{2}}$$

The receiver Bob randomly selects |V > or |R > each time to measure the quantum signal transmitted by the sender. Among them, |V > and |R > are expressed in the language of quantum mechanics as follows:

8 $$|V> =|1>$$

9 $$|R> = \frac{|0>-|1>}{\sqrt{2}}$$

When the B92 protocol starts to be executed, Alice randomly prepares a string of binary bits selects the polarization state of the coded base modulated photon, and sends the modulated photon string to the receiver Bob in sequence according to a certain time interval, and then Bob Each photon randomly selects a measurement base for measurement. Next, Bob tells Alice through the classic channel the location of the determined measurement result but does not transmit the selected measurement base string. Theoretically, if Bob measures “↑”, Alice must send “↗”; if Bob measures “↘”, Alice must send “→”.

**Figure 2:**
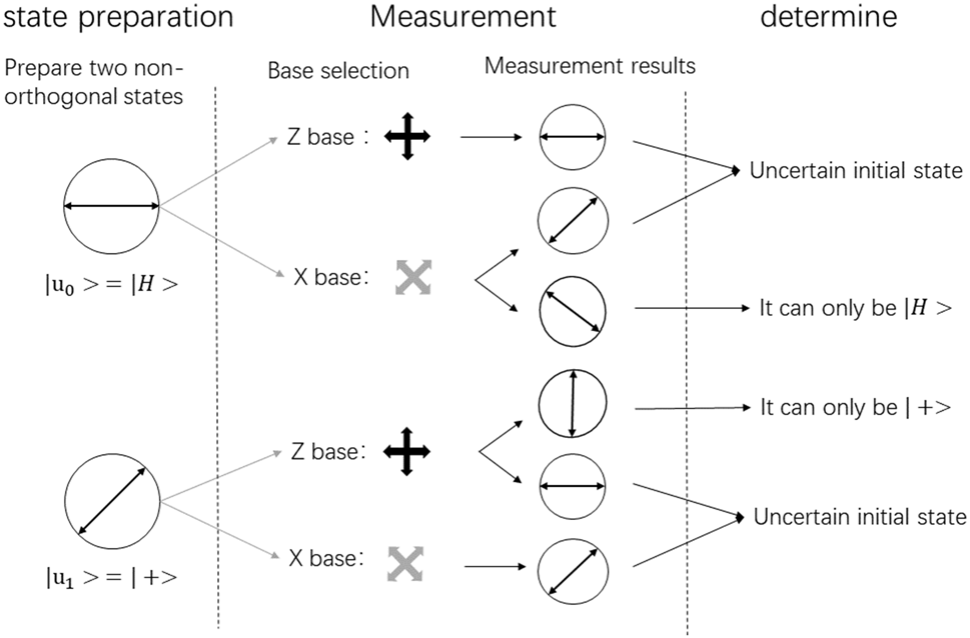
Schematic diagram of the B92 two-state protocol principle.

As shown in Fig. 2 below, this correspondence has a 25% probability of occurring relative to all events, and the measurement results can be obtained, that is, the efficiency of the B92 protocol is only 25%, which is only half of that of BB84.

Subsequently, the sender Alice, and the receiver Bob retain all the qubits and measurement bases that have obtained certain measurement results. The rest indicate that a transmission error (*Quantum Error*) has occurred and can be discarded. Next, Alice and Bob randomly select some bits from the original key for eavesdropping detection. If the error rate is less than the threshold, proceed to the next step; otherwise, it is considered that there is eavesdropping and the protocol is terminated.

Alice and Bob further entangle and amplify the confidentiality of the negotiated key and finally obtain an unconditional security key^18,19^. As shown in Table 1, when Alice sends 0°, if Bob uses the Z base to receive 0°, the result cannot be retained, because Alice may send 45°; when Alice sends 45°, Bob uses the X base to receive it 45°, the result cannot be retained, because 0° can also become 45°; when Alice sends 45°, Bob uses the Z basis to receive 90°, the result can be retained because 0° cannot become 90°.

**Table 1:** Schematic diagram of B92 quantum key distribution results.

Alice generates a random sequence	0	1	1	0	1	0	0
Alice’s chosen polarization state	→	↗	↗	→	↗	→	→
The detection base selected by Bob	$+$	$\times$	$+$	$\times$	$+$	$+$	$\times$
Bob’s measurements	→	↗	↑	↘	↑	→	↘
Whether to retain	N	N	Y	Y	Y	N	Y
Final results			1	0	1		0

If the attacker Eve operates on the qubits |φ > photons and |$\phi$> photons, errors will inevitably be introduced. According to the correlation of Alice and Bob's measurement results, they can check the existence and existence of eavesdropping attacks during eavesdropping detection. Therefore, the B92 protocol is unconditionally safe, and its security is also guaranteed by the basic principles of quantum theory.

Under ideal conditions, that is, without the influence of the attacker Eve's eavesdropping and channel noise disturbance, the probability that Bob obtains a certain measurement result of |φ > or |$\phi \hspace{0.17em}$> each time is:

10 $${p}_{C}=\frac{1-{||\langle \varphi |\phi \rangle ||}^{2}}{2}$$

The probability of error is:

11 $${p}_{f}=1-{p}_{C}=\frac{1+{||\langle \varphi |\phi \rangle ||}^{2}}{2}$$

### Integration method of quantum communication and logistics information system

To form a *quantum logistics big data cloud platform*, it is first necessary to integrate quantum communication technology with traditional logistics parks, rely as much as possible on existing computer rooms, optical fiber, and other communication infrastructure to build a QKD network, and add QKD-related equipment. According to the application of existing information system keys in the highway logistics and transportation industry, the integration of quantum communication technology and information systems can be divided into the following two situations, which will be presented later.

Firstly, we need to understand that the core goal of integrating quantum communication and logistics information systems is to form a quantum logistics big data cloud platform. This requires us to integrate quantum communication technology with traditional logistics parks, relying as much as possible on existing computer rooms, optical fibers, and other communication infrastructure to build a Quantum Key Distribution (QKD) network and add QKD-related equipment.

In existing logistics parks, there is already a complete set of communication infrastructure, including computer rooms, optical fibers^20^, etc. We need to add QKD-related equipment to these infrastructures to realize quantum communication functions. This may require us to upgrade some hardware and optimize software to adapt to the characteristics of quantum communication.

In the highway logistics and transportation industry, there is already a mature information system. We need to integrate these systems with quantum communication technology to achieve more efficient and safer data transmission and processing. This may require us to modify and upgrade the existing information system to adapt to the characteristics of quantum communication.

According to the application of existing information system keys in the highway logistics and transportation industry, the integration of quantum communication technology and information systems can be divided into the following two situations.

The first situation is that we can directly add quantum communication functions to the existing information system to realize quantum encryption and decryption of data. The advantage of this method is that it can directly utilize the existing information system without the need for large-scale transformation. However, this may also bring some challenges, such as how to add quantum communication functions without affecting the performance of the existing system.

The second situation is that we can build a brand new quantum information system to run in parallel with the existing logistics information system. The advantage of this method is that it can fully utilize the advantages of quantum communication to achieve more efficient and safer data transmission and processing. However, this may also require more investment, including the purchase of hardware equipment and the development of software systems.

In summary, the integration of quantum communication and logistics information systems is a complex process that requires us to understand the basic principles of quantum communication and combine the actual needs of the logistics industry to make appropriate design and planning. Only in this way can we truly realize the construction of the quantum logistics big data cloud platform and promote the development of the logistics industry.

#### Integration with existing encryption machines or key management module information systems

For the private information of both parties to the transaction, the original system was encrypted using traditional asymmetric methods; for some military goods or special chemical goods, the entire transportation chain is encrypted. For these encrypted information modules, there is no need to transform the business application layer of the information system. We only need to build a *QKD network* to achieve true random distribution of quantum keys, and by making relevant modifications to encryption equipment or routing equipment and adding interfaces that can transmit quantum keys in real-time, users can easily use quantum keys to encrypt and decrypt business system data. In addition, the existing key management module can also be modified and the keys can be uploaded to the quantum key cloud service platform to achieve unified management of existing keys and quantum keys.

Firstly, we need to understand that the integration of existing encryption machines or key management module information systems is a crucial step in the process of integrating quantum communication and logistics information systems. This mainly involves the following aspects.

In the original system, we encrypted the private information of both parties to the transaction using traditional asymmetric methods. Although this method can protect the security of information to a certain extent, its security may be greatly reduced when facing the threat of quantum computing. Therefore, we need to introduce quantum communication technology and use the Quantum Key Distribution (QKD) network to achieve the true random distribution of quantum keys, and thus enhance the security of information.

For some military goods or special chemical goods, we need to encrypt the entire transportation chain. This requires us to add the function of quantum communication in the existing information system to realize the quantum encryption and decryption of data. This may require us to make relevant modifications to the existing encryption equipment or routing equipment and add interfaces that can transmit quantum keys in real-time.

In addition to encrypting data, we also need to manage keys. We can modify the existing key management module, upload the keys to the quantum key cloud service platform, and achieve unified management of existing keys and quantum keys. This can not only improve the efficiency of key management but also further enhance the security of the system.

#### Integration with information systems without encryption machines or key management modules

In the process of integrating quantum communication and logistics information systems, for information systems without encryption machines or key management modules, we can adopt the following methods:

We can install a quantum key cloud terminal on the transport vehicle, or build the SDK of the quantum key cloud terminal before the vehicle leaves the factory. In this way, we can directly use quantum encryption-related equipment to build a QKD network. The advantage of this method is that it can directly use the existing vehicle equipment without additional hardware installation^21,22^. However, this may also bring some challenges, such as how to ensure the security and stability of the quantum key cloud terminal.

Distribution and management of keys: We can realize the distribution and management of keys through the quantum key cloud service platform and the logistics big data platform. This can not only improve the efficiency of key management but also further enhance the security of the system. In addition, we can also provide quantum keys to encrypt and decrypt business data, such as vehicle location, driver identity, and cargo information.

Encryption and decryption of business data: By using quantum keys, we can encrypt and decrypt business data. This can effectively protect the security of data and prevent data from being stolen or tampered with during transmission. At the same time, due to the randomness and uniqueness of quantum keys, even if the key is leaked, it will not have a significant impact on the security of the system.

In summary, for information systems without encryption devices or key management modules, we can deploy quantum key cloud terminals, build QKD networks, realize the distribution and management of keys, and encrypt and decrypt business data to achieve the integration of quantum communication and logistics information systems. This can not only improve the security of the system but also improve the operational efficiency and service quality of the logistics industry. However, this also requires us to fully consider various possible challenges and problems in the implementation process and develop reasonable solutions. Only in this way can we truly realize the construction of the quantum logistics big data cloud platform and promote the development of the logistics industry.

### Quantum communication network construction strategy

Due to current quantum signals mainly using photons to transmit signals and content, there are two main network construction strategies for quantum communications. The first is a *relatively high-cost independent optical fiber method* that needs to be used between all units that need to communicate. Brand-new optical fiber lines are expensive and have a low usage rate when quantum communication is not yet fully popularized. The second type is a lower-cost *optical fiber wavelength division multiplexing method*, which is combined with traditional communication methods and does not require independent fiber. The original optical fiber lines are still used to complete the transmission of optical signals, which has a high utilization rate. However, the disturbance noise between multiple signals cannot be completely removed at present, which will have a certain impact on the transmission of quantum signals and will affect the amplitude and angle of quantum spin. Changes occur, that is, erroneous transmissions. The current quantum error correction mechanism can solve this problem to a certain extent, but the current technology can only solve the situation when an error occurs in a single quantum and needs to continue to develop.

**Figure 3:**
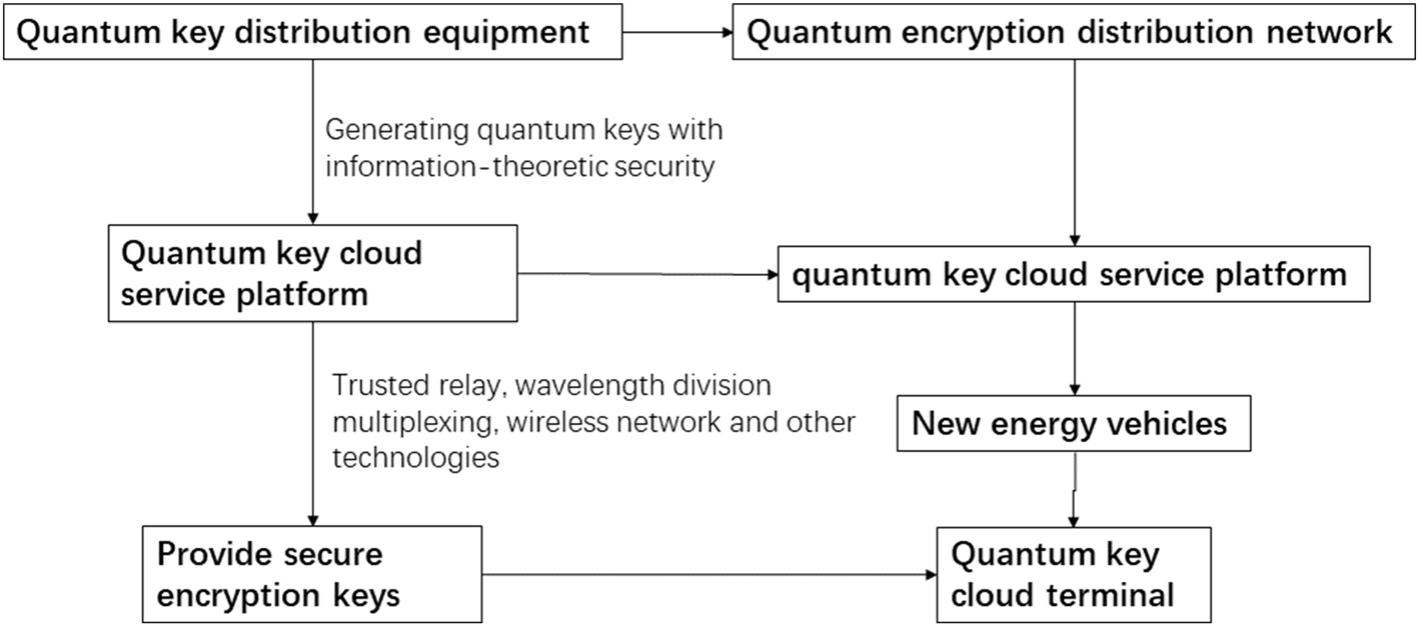
Schematic diagram of signal transmission construction of Quantum Highway Logistics Big Data Platform.

This structure adopts the second method^23^ and combines it with other methods, such as *key trusted relay technology*, *wavelength division multiplexing technology*, *wireless network*, etc., to complete the construction of the quantum highway logistics big data platform, as shown in Fig. 3.

## Cloud service logistics information system

### Overall system architecture

**Figure 4:**
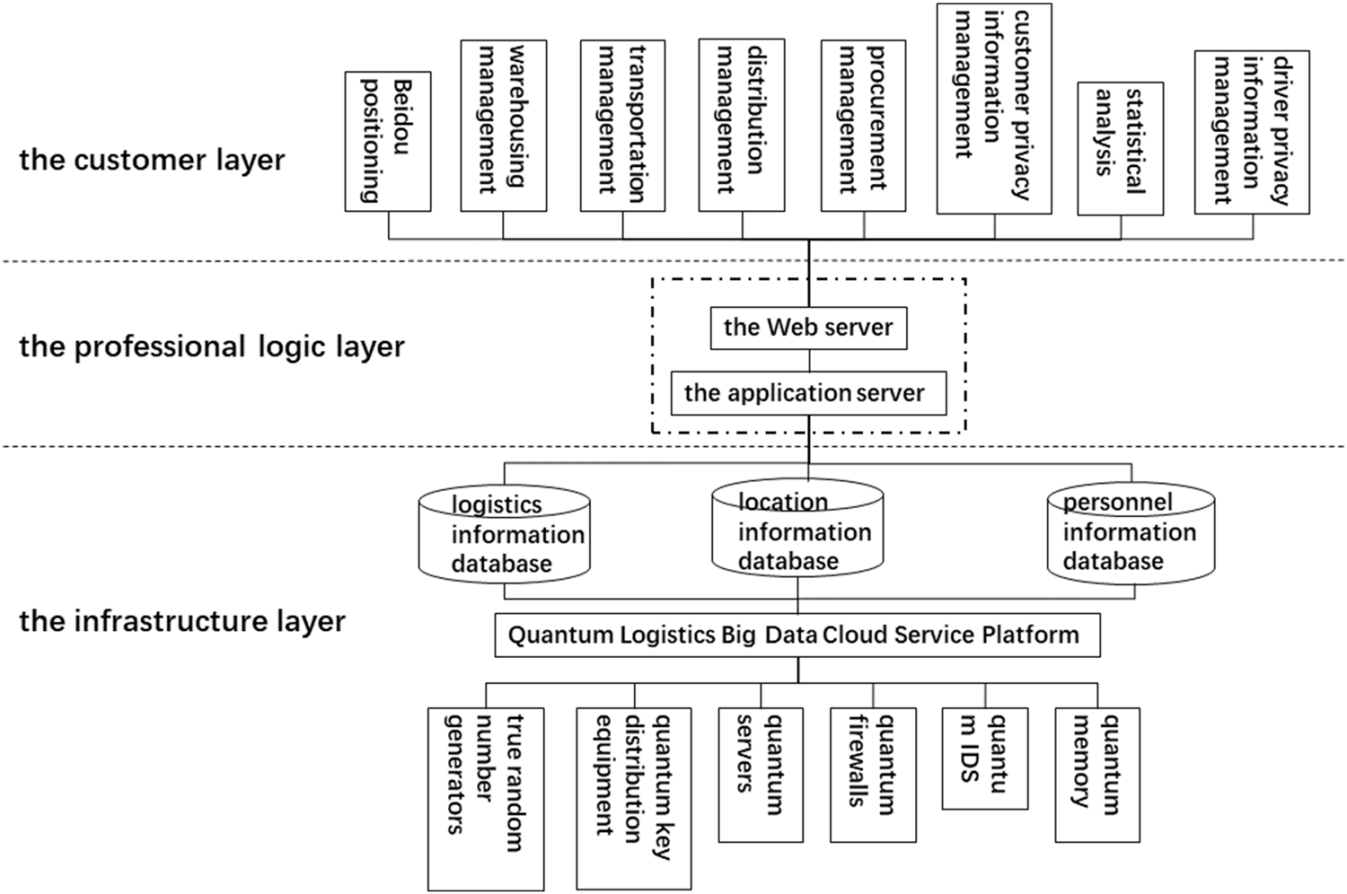
Overall architecture diagram of cloud service logistics information system.

As shown in Fig. 4, the overall architecture of the *cloud service logistics information system* is mainly divided into three layers: the customer layer, the professional logic layer, and the infrastructure layer^24^. Each layer contains specific components that contribute to the overall functionality of the service platform.

The customer layer: This layer is positioned at the top of the diagram and includes various management modules. These modules include bidirectional positioning management, warehousing management, transportation management, distribution management, procurement management, and the analysis and management of customer privacy disclosure agreements. These modules interact directly with the “Web server” in the professional logic layer to meet customer needs and provide services. Use the browser to open the connection manage it, and connect to the Web server of the profession logic layer respectively. The professional logic layer also includes an application server, which is connected upward to the Web server and downward to three important databases in the infrastructure layer.

The professional logic layer: This layer is located in the middle section of the diagram and contains the “Web server” and the “application server”. The main role of this layer is to serve as an intermediary between the customer layer and the infrastructure layer, processing and transmitting information. The “Web server” is responsible for handling requests from the customer layer and forwarding these requests to the “application server” for processing. The “application server” is responsible for handling these requests and interacting with the databases in the infrastructure layer to retrieve or update data.

The infrastructure layer: This layer is positioned at the bottom of the diagram and houses the logistics information database, location information database, and personnel information database. These databases store all the data needed for the platform to operate, including logistics information, location information, and personnel information. Meanwhile the database should adopts an incremental backup method and be backed up once a week. It needs to be backed up offsite. In addition, this layer is connected to various elements such as quantum generators, quantum key distribution equipment, etc., indicating that it is a complex data processing and storage system.

The entire structure is labeled “Quantum Logistics Big Data Cloud Service Platform” at the bottom, indicating that this entire structure is part of this platform. This platform uses quantum technology and big data technology to provide efficient and secure logistics services to customers.

The entire system is ultimately managed uniformly by the Quantum Logistics Big Data Cloud Service Platform, using quantum technology and big data technology to provide efficient and secure logistics services to customers, ensuring seamless communication and efficient transmission of data while ensuring data security.

### System functions

The Quantum Logistics Big Data Cloud Service Platform should include 8 main functions, as shown in Fig. 4.

Bidirectional positioning management: This function corresponds to China’s original Beidou positioning subsystem, providing map query services and real-time positioning services. This means that no matter where the goods are, they can be tracked and located in real-time through this system, ensuring the safety and timely delivery of the goods.

Warehousing management: This function provides real-time updated names, types, quantities, etc. of goods stored in the warehouse information. This is crucial for maintaining inventory management and avoiding inventory shortages or surpluses.

Transportation management: This function provides the transportation status of each item of goods, such as courier information, current location information, traceability, etc. In this way, no matter where the goods are, their status can be tracked in real-time, ensuring the safety and timely delivery of the goods.

Distribution management: This function can automatically allocate delivery personnel according to the jurisdiction where the goods are located, making the distribution work efficient and convenient, allowing the recipient to receive the goods faster, and save time. This is crucial for improving customer satisfaction and maintaining efficient operations.

Procurement management: This function can record and analyze all purchased goods in this system and their key information, including purchase time, purchase object, docking person, name of purchased goods, risk level of goods, etc. This is crucial for maintaining supply chain management and avoiding supply chain interruptions.

Customer privacy information management: This function complies with the national real-name system regulations, which require the collection of customers’ names, phone numbers, ID numbers, home addresses, and other information to ensure that the overall process is legal and compliant. At the same time, it specifically records and stores customers’ personal information to ensure its absolute security, customer privacy will never be leaked.

Statistical analysis: This function will automatically count the cargo transportation throughput and other information in each region based on the shipping address, receiving address, transportation time, and other information to analyze whether there is a need to increase or decrease express delivery in the region. Personnel and courier points to save costs. This is crucial for optimizing operations and reducing operating costs.

Driver privacy information management: This function is used to record the basic personal information of company employees and manage it separately from customer privacy information, which can more effectively protect the security of these two modules and avoid being completely exposed to ransomware expose all information.

In addition, the logistics big data platform integrates data between multiple heterogeneous logistics platforms, has a distributed logistics service resource pool, and is compatible with collaborative operations. This means that no matter which logistics platform’s data, it can be integrated and analyzed through this platform, thereby maximizing the use of data, and improving operational efficiency and customer satisfaction. In summary, this Quantum Logistics Big Data Cloud Service Platform is a powerful tool that can help logistics companies improve efficiency, reduce costs, improve customer satisfaction, and achieve sustainable development.

## Application plan of highway logistics office information system based on quantum communication technology

### Overall architecture of quantum secure communication application solution

**Figure 5:**
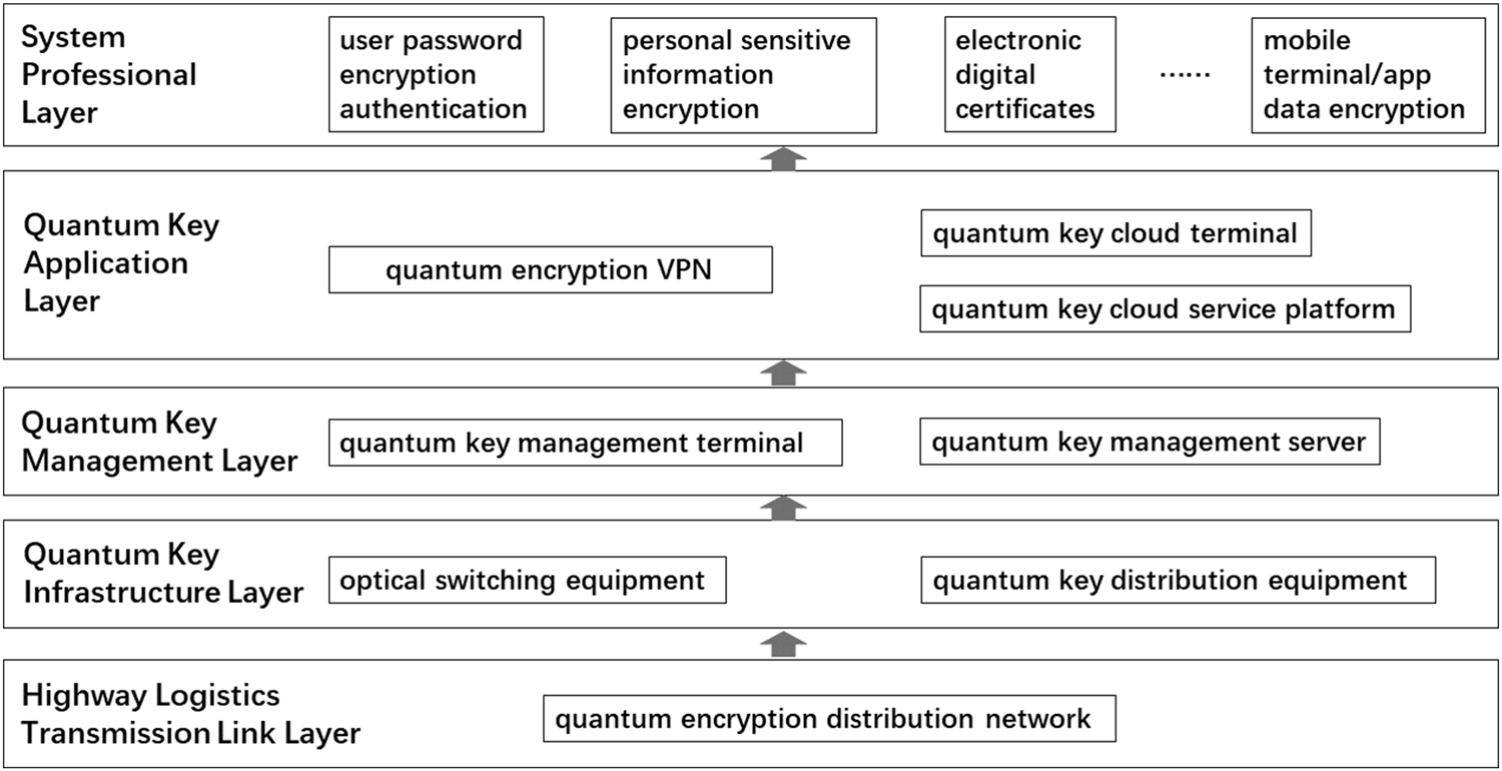
Overall architecture of quantum secure communication application solution.

The road logistics office information system application solution based on quantum communication technology will be consistent with the existing road logistics transportation network infrastructure in terms of facility deployment and will be consistent with the existing road logistics in terms of resource management and use of quantum key distribution^25^. The top-down business management method of the transportation office information system is integrated. The overall architecture of this solution is shown in Fig. 5, which includes the system professional layer, quantum key application layer, quantum key management layer, quantum key infrastructure layer, and highway logistics transmission link layer from top to bottom.

#### Highway logistics transmission link layer

*The highway logistics transmission link layer* is constructed from the quantum encryption distribution network, including the backbone transmission network, access transmission network, and convergence transmission network, all of which are wavelength division multiplexed with traditional network routes to efficiently complete the transmission of quantum encryption information and keys. The logistics big data platform connects the quantum encryption distribution network and the quantum key cloud service platform, which provides service interfaces for the SAAS layer upwards and manages quantum application terminals downwards^26^.

#### Quantum key infrastructure layer

The main types of equipment in *the quantum key infrastructure layer* include optical switching equipment, which is used to complete the switching of optical links, and quantum key distribution equipment, which is used to efficiently generate and distribute quantum keys. Under the control of the quantum key management layer, the quantum key infrastructure layer will cooperate with optical path switching and quantum key distribution most efficiently to complete the overall function.

#### Quantum key management layer

*The quantum key management layer* mainly includes two terminals, namely the quantum key management terminal, which is used to provide point-to-point quantum keys to the quantum key application layer, and the quantum key management server, which is used to realize the unification of keys and blending.

#### Quantum key application layer

*The quantum key application layer* includes quantum encryption VPN to achieve quantum encryption secure communication; the quantum key cloud terminal, placed on the vehicle, allows the administrator to grasp the vehicle's location information promptly while ensuring key information (such as sender Information, recipient information, cargo information, geographical location information, departure, and destination information, etc.) are safe and without leakage; the quantum key cloud service platform uses technologies including but not limited to trusted relay technology, B92 protocols, wireless networks, and optical lines are multiplexed to generate end-to-end shared keys in the quantum encryption distribution network and provide secure encryption keys through the interface.

*The quantum key application layer* applies for quantum keys from the quantum key management layer, receives data from the system business layer, and completes data encryption and decryption at the same time.

#### System professional layer

The functions that need to be used are planned in the professional layer of the system, including user password encryption authentication, personal (customer/driver) sensitive information encryption, electronic digital certificates, mobile terminal/app data encryption, and other functions, and can be optimized as needed, as much as possible all covered.

### Quantum secure communication logic architecture

**Figure 6:**
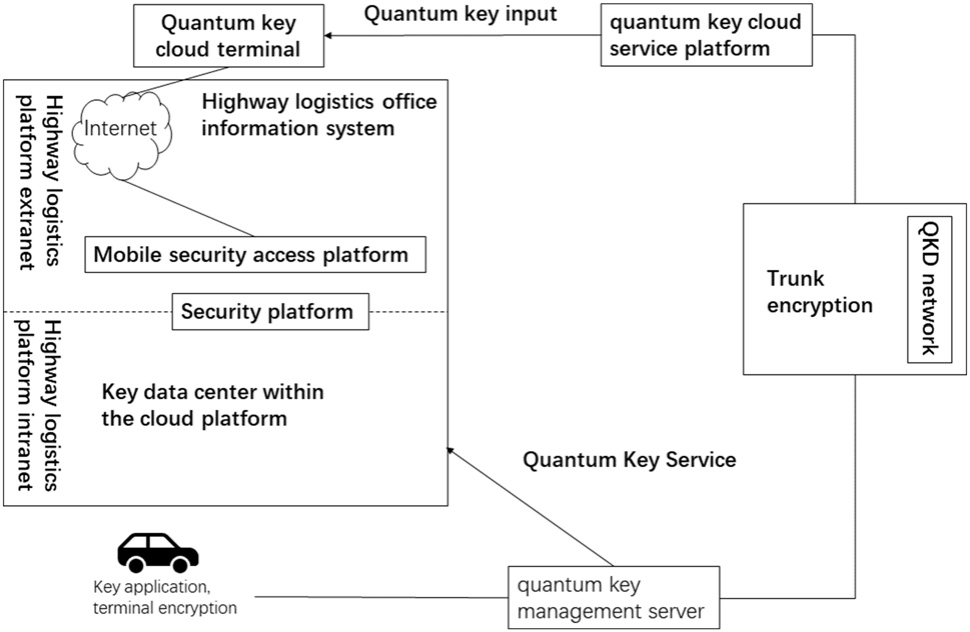
Quantum secure communication logical architecture diagram.

The first step in applying quantum communication technology is to build a *quantum key distribution (QKD) network* and carry out applications on this basis^27^. The logical architecture diagram of the highway freight information system application solution based on quantum communication is shown in Fig. 6.

The QKD network of the highway transportation office information system allows the system's professional layer to obtain a steady stream of quantum keys (one-time encryption) from the network; the quantum key management server can uniformly manage quantum encryption keys and provide services for the entire office. The information system provides secure quantum key services to achieve secure encryption of all terminal information in the system; the quantum key cloud service platform can provide quantum key charging services for mobile devices to solve the problem of mobile users' (drivers) safety issues during use.

#### Quantum key management server

This solution is based on the Quantum Key Distribution (QKD) network, which generates currently absolutely secure quantum keys and transmits them to the quantum key management server. The quantum key management server provides unified quantum encryption key services to all end users based on the evaluation of the number of key services in the overall system. Each subsystem can directly use traditional symmetric key encryption, or it can also encrypt key contents such as digital certificates. Perform secondary encryption. The keys generated by the quantum key management server are based on a random number generation algorithm seeded with security and high system entropy in the cryptographic machine, thus protecting the keys from being deciphered by attackers. The quantum key is protected by the security mechanism of the hardware. The plaintext of the quantum key is only used for quantum cryptography operations inside the quantum cryptography machine and will not leave the security boundary of the quantum cryptography machine hardware^28^.

Quantum key distribution (QKD) is a technique that allows two parties to securely exchange encryption keys using quantum physics. QKD requires special devices such as a laser source, which emits single photons or weak pulses of light, and another optical device, such as a beam splitter or a phase modulator, to implement it. The laser source and the optical device are connected to each other by a quantum channel, such as a fiber optic cable or free space. The quantum channel allows the transmission of quantum states, which carry the information of the encryption keys. The two parties also need classical communication channels, such as the internet or telephone lines, to verify and authenticate the keys. QKD can be integrated into current digital infrastructure by using existing network components, such as routers, switches, and multiplexers, to support both quantum and classical signals. However, this integration poses some challenges, such as ensuring the compatibility and interoperability of different QKD systems and protocols, protecting the quantum channel from noise and eavesdropping, and optimizing the performance and efficiency of QKD in realistic scenarios.

The unified management of quantum keys through the quantum key management server can effectively ensure that all work between users at all levels in the system is carried out safely and efficiently. Setting up the quantum key management server in this application has the following three advantages:

1. *User permissions can be combined to achieve hierarchical management of quantum keys.* The quantum key management server dynamically allocates corresponding quantum key resources according to the security level of the system business, business conditions, and the user's use of quantum keys;
2. *Different quantum keys can be used according to different services.* This further achieves safe isolation between business data of different subsystems, while ensuring regular updates of quantum keys for key businesses, improving the security of the overall system;
3. *Compatible with traditional keys and quantum keys.* Realize the integrated management of multiple keys and gradually replace traditional keys with quantum keys to facilitate the smooth transition of business.

#### Quantum key cloud service platform

Taking into account the security issues of the system's mobile terminal users (drivers) when using the system, a quantum key cloud service platform is designed in this plan, which can realize quantum key charging for all mobile terminals and control the terminal's quantum key^29,30^. Unified management of keys and more secure provision of services such as login authentication and data storage to mobile users.

Quantum Key Distribution (QKD) equipment completes the transmission of quantum state optical signals between communicating parties, generates quantum keys with information theory security, and uploads the keys to the Quantum Key Cloud Service Platform; Quantum Key Cloud Service Platform realizes end-to-end shared key generation in the network through trusted relay and other technologies, completes functions such as network management and key management, and provides secure encryption keys for quantum encryption application equipment through application interfaces.

The built-in quantum key cloud terminals in each vehicle of this solution are connected through the quantum key cloud service platform. Through the multiplexing and transmission of quantum channels and classical optical channels, it can effectively save the fiber core pipeline resources required for the deployment of quantum secure communication networks. Utilize the existing optical communication network to achieve the goal of economical and efficient network construction. There is no need to deploy dedicated secure channels or optical fiber channels on the highway. In this way, QKD secure communication between each vehicle can be achieved.

This solution uses a one-time pad, and the quantum keys are independent of each other. If the mobile terminal is damaged, the vehicle only needs to be logged off individually through the quantum key cloud service platform, without affecting the operation of other vehicles.

### Constructing a quantum highway logistics big data platform

A quantum highway logistics big data platform is a system that uses quantum computing and communication technologies to collect, process, analyze, and share large-scale data related to the transportation of goods and services^5^. Based on the two major frameworks and contents described earlier, the author will use the following steps to construct a quantum highway logistics big data platform:

1. Designing a quantum network architecture that connects the logistics nodes, such as warehouses, vehicles, ports, and customers, using quantum key distribution and quantum repeaters for secure and efficient data transmission;
2. Developing a quantum database that stores and manages the logistics data, such as inventory, demand, supply chain, routing, and delivery status, using quantum error correction and quantum encryption for data protection and integrity;
3. Implementing a quantum algorithm that performs advanced analytics on the logistics data, such as optimization, simulation, forecasting, and decision-making, using quantum machine learning and quantum optimization for enhanced performance and accuracy;
4. Integrating a quantum interface that enables the interaction between the platform and the users, such as logistics managers, operators, regulators, and consumers, using quantum visualization and quantum feedback for user-friendly and interactive data presentation and manipulation.

## Conclusion

This paper combines the relevant content of quantum communication applications and logical architecture, as well as the overall architecture of the highway logistics and transportation office information system, and proposes a quantum communication-based highway freight information system application scheme. Both parties in the communication use symmetrical quantum key distribution. The key is used to encrypt and decrypt information, and the quantum key management server and quantum key cloud service platform are used to achieve unified management of quantum keys and secure encryption of vehicle data transmission, thereby ensuring the security of road freight information system data transmission. In the next step, after the construction of the quantum communication network is fully completed, all systems can use quantum key encryption, thereby improving the overall security of the logistics and transportation industry.

## Data Availability

The datasets generated and/or analyzed during the current study are not publicly available due confidentiality requirements of the school but are available from the corresponding author on reasonable request.
